# Keeping Excitation–Inhibition Ratio in Balance

**DOI:** 10.3390/ijms23105746

**Published:** 2022-05-20

**Authors:** Sergei Kirischuk

**Affiliations:** Institute of Physiology, University Medical Center, Johannes Gutenberg University, 55128 Mainz, Germany; kirischu@uni-mainz.de

**Keywords:** neurological disorders, genetic mouse models, binomial model of synaptic transmission, readily releasable pool, release probability, quantal size

## Abstract

Unrelated genetic mutations can lead to convergent manifestations of neurological disorders with similar behavioral phenotypes. Experimental data frequently show a lack of dramatic changes in neuroanatomy, indicating that the key cause of symptoms might arise from impairment in the communication between neurons. A transient imbalance between excitatory (glutamatergic) and inhibitory (GABAergic) synaptic transmission (the E/I balance) during early development is generally considered to underlie the development of several neurological disorders in adults. However, the E/I ratio is a multidimensional variable. Synaptic contacts are highly dynamic and the actual strength of synaptic projections is determined from the balance between synaptogenesis and synaptic elimination. During development, relatively slow postsynaptic receptors are replaced by fast ones that allow for fast stimulus-locked excitation/inhibition. Using the binomial model of synaptic transmission allows for the reassessing of experimental data from different mouse models, showing that a transient E/I shift is frequently counterbalanced by additional pre- and/or postsynaptic changes. Such changes—for instance, the slowing down of postsynaptic currents by means of immature postsynaptic receptors—stabilize the average synaptic strength, but impair the timing of information flow. Compensatory processes and/or astrocytic signaling may represent possible targets for medical treatments of different disorders directed to rescue the proper information processing.

## 1. Introduction

Despite the accumulating data on factors, including genetic, immune, environmental, and associated with neurodevelopmental and psychiatric disorders, the underlying mechanisms leading to the manifestation of symptoms remain poorly understood [1,2]. As the anatomy of the brain seems to not be dramatically changed in the case of many brain disorders, recent data showing that some unrelated genetic mutations can result in the manifestation of quite similar phenotypes—for instance, social behavioral deficits—suggest that the main cause of symptoms is the way neurons communicate, i.e., a disturbance of neuronal network activity. It was suggested that both an increase in neuronal activity, which makes the network noisier, and a decrease in the activity, which would make the network too quiet, may potentially lead to pathological symptoms later in development [3]. The dominating working hypothesis is that a transient change in the excitation–inhibition (E/I) balance may underlie the development of some neurodevelopmental and psychiatric diseases (for a recent review, [4]). Indeed, in some cases, significant improvements in symptoms can be achieved by systemic pharmacological changes in GABAergic transmission [5,6].

In the frame of the E/I imbalance hypothesis, a pathological shift of the physiological value can occur as a result of changes in the glutamatergic and/or GABAergic drive. Recently developed optogenetic tools allow for the selective activation or inhibition of a specific population of neurons in the CNS in vivo [7]. The selective optogenetic activation of excitatory pyramidal neurons in the medial prefrontal cortex (mPFC) acutely impairs the explorative and social behavior of mice, showing that a shift in the E/I ratio towards excitation is sufficient to acutely influence mouse behavior [8]. A further study from this group demonstrated that the specific activation of inhibitory interneurons only or the selective inhibition of pyramidal cells in the mPFC is sufficient to rescue social behavioral deficits in a genetic mouse model of autistic spectrum disorder (ASD), supporting the hypothesis that the elevated E/I ratio in the adult animals might explain the observed behavioral changes [9]. These results show that an acute E/I shift towards excitation is capable of inducing ASD-like symptoms and that shifting the E/I ratio towards inhibition is sufficient to acutely alleviate the deficits in the adult mice. In addition, it has recently been shown that a transient selective hyperactivation of cortical pyramidal neurons during early postnatal development, namely between postnatal days (P) 4–14, is sufficient to cause a decreased social interaction and increased grooming behavior in adult animals. In vitro recordings from pyramidal neurons in the prefrontal cortex confirmed the E/I shift towards excitation in adult animals, indicating that a relatively short period of externally driven hyperactivity can (1) induce a long-lasting shift of the E/I ratio and (2) lead to the manifestation of behavioral deficits that are only evident in adult animals [10].

It is worth mentioning, however, that, in the above studies, a non-physiological stimulation protocol has been applied. Optogenetically driven excitation/inhibition is (i) relatively long-lasting, especially when compared with the duration of a single action potential, and (ii) distributed across a broad population of neurons. Deviations from the physiological level of the E/I ratio in both directions have been observed in many mouse models of neurodevelopmental diseases. However, this imbalance is quite frequently only transient. In tuberous sclerosis factor 1 heterozygote mice (Tsc1+/−), an ASD mouse model, hyperexcitability and epileptic seizures occur only early postnatally (<P19, [11]). In the mPFC of Tsc2+/− mice, another ASD mouse model, an elevated E/I ratio has been observed early postnatally (<P20) but not later (P > 30). In this case, the increased glutamatergic drive was compensated for by an elevated GABAergic transmission, thus re-balancing the overexcited neuronal network [12]. Using four different mouse ASD models, Antoine et al. [13] reported an elevated E/I ratio in the somatosensory cortex during early postnatal development (P15–19). Further experiments performed in vitro and in vivo, however, revealed that the observed shift in the E/I ratio towards excitation does not result in network hyperactivity. The elevated E/I balance rather serves as a compensatory factor, stabilizing the sensory-input-induced firing rate of pyramidal neurons in the somatosensory cortex in those genetically modified mice in vivo. Consequently, the authors conclude that a transiently elevated E/I ratio is not a destabilizing factor but rather represents a homeostatic mechanism that stabilizes the neuronal network functioning within a critical “instable” period of development, presumably induced by a genetic deficiency. If this hypothesis is true, then it is reasonable to suggest that the elevated E/I balance would be stabilized later in development and that the proper functioning of the cortical networks will be achieved by other physiological means.

As the E/I ratio is only a one-dimensional parameter, it is rather unlikely that its imbalance is the only cause of so many different neurological disorders [14]. Indeed, many other changes, including anatomical, morphological, and functional alterations, have to be considered when investigating the causes of neurological deficits. This review will describe experimental data from several genetic mouse models of neurodevelopmental and psychiatric disorders focusing on glutamatergic and GABAergic synaptic transmission, and, in turn, the E/I ratio, at the single cell level. Using the binomial model of phasic synaptic transmission, it will be shown that, usually, several counteracting physiological alterations are in progress during early development, and that these processes are capable of stabilizing the mean E/I ratio, but fail to support the required temporal precision of information transfer.

## 2. E/I Balance in the Frame of Binomial Model of Synaptic Transmission

In the frame of the binomial model of synaptic transmission, the strength of the synaptic input, i.e., the mean amplitude or, more precisely, the charge of the evoked response elicited by a single action potential, is described as the product between the number of synaptic contacts, the mean release probability (Pv), i.e., the probability that a single synapse releases at least one vesicle, and the quantal size, i.e., the mean amplitude or charge of the postsynaptic response elicited by the content of a single presynaptic vesicle [15]. The mean amplitude or charge of postsynaptic currents recorded in the presence of tetrodotoxin, a blocker of voltage-gated Na+ channels, so-called miniature excitatory and inhibitory postsynaptic currents (mEPSCs and mIPSCs, respectively), are frequently used as a functional estimate of the quantal size. When the m(E/I)PSC frequency is low—for example, early postnatally—the mean amplitude or charge of spontaneous excitatory and inhibitory postsynaptic currents (sEPSCs and sIPSCs), i.e., responses recorded in an artificial cerebrospinal solution without the addition of tetrodotoxin, is used. However, the mean amplitude of sPSCs depend on the network activity and might also be a multi-quantal amplitude.

It is important to mention that, during development, the quantal size, originally supposed to be constant, is variable, and that its charge transfer depends on several physiological parameters, including the amount of released neurotransmitters (presynaptic vesicle filling [16]), the number of postsynaptic receptors, the type(s) of postsynaptic receptors (for example, slow or fast type [17]), and the driving force for the ions that can flow through the open channels (for instance, Cl^-^ for GABA_A_Rs). The latter parameter represents the difference between the resting membrane potential and the reversal potential for the permeable ion(s). Both potentials are developmentally regulated. In the case of GABAergic transmission, the action of GABA even demonstrates a developmental change in polarity, namely from depolarizing to hyperpolarizing [18,19].

The number of functional synaptic contacts is determined by the dynamic balance between synaptogenesis and synaptic pruning (for review, [20,21,22]). The real (current) number of anatomical contacts can reliably be counted only in fixed tissue. Moreover, different synapses may possess a different number of synaptic vesicles that are ready for release, called the readily releasable pool of presynaptic vesicles (RRP). This parameter can be measured without a tissue fixation and is frequently used as a functional estimate of the number of release sites [23,24]. Two main approaches have been developed to obtain the RRP size in vitro: hyperosmotic solution application [23] or by the high-frequency electrical stimulation of synaptic inputs [24,25]. It is worth mentioning that the latter RRP estimate represents an action potential- and presynaptic Ca^2+^-dependent vesicle pool, whereas the hyperosmotic solution-induced depletion of RRP is Ca^2+^-independent [23]. Moreover, the RRP size, i.e., the number of presynaptic vesicles available for release, is not constant and is influenced by the level of presynaptic activity. A single presynaptic action potential elicits a release of several vesicles, thus reducing the number of those remaining in the RRP. The “missing” vesicles will be replaced by new ones from the reserve pool via RRP replenishment or a refilling mechanism. The latter process is sensitive to the activity level; for instance, the RRP replenishment rate is sped up by a higher presynaptic Ca^2+^ concentration [26]. Thus, both the RRP size and the rate of RRP replenishment are required for the estimation of the functional RRP size and, in turn, the current synaptic strength.

The presynaptic release probability (Pv) is the parameter that is the most difficult to estimate. More frequently, the amplitude of the evoked response is used to obtain the mean release probability value, provided that the RRP size is available, which is not always the case [27]. Frequently, the paired-pulse ratio (PPR) is used as an estimate of the presynaptic release probability. In this case, two stimuli separated by a short interstimulus time interval are applied. The ratio of the mean amplitude of the second evoked response to the mean amplitude of the first response may be considered to report an approximation of the presynaptic release probability. The higher the release probability, the more vesicles that will be released by the first stimulus, resulting in a larger amplitude of the first postsynaptic response and a smaller number of vesicles remaining in the RRP. The latter leads to a smaller second response. Thus, the higher the PPR, the higher the presynaptic release probability [28,29]. However, it has to be kept in mind that PPR is a dynamic parameter. In addition to “steady-state” changes in Pv, this parameter is also sensitive to both pre- and postsynaptic short-term changes; for example, a desensitization of postsynaptic receptors or presynaptic short-term facilitation.

**Figure 1:**
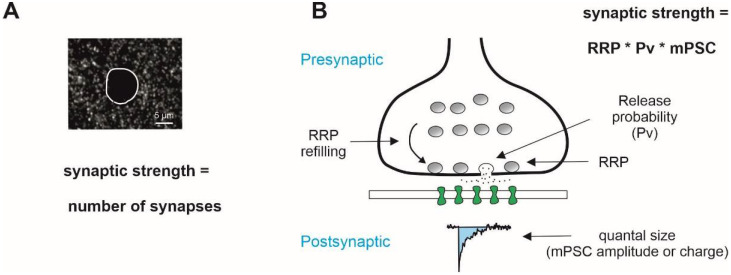
General mechanisms that determine the strength of synaptic transmission. (**A**) Number of synaptic contacts visualized immunohistochemically is frequently taken as an estimate of the strength of synaptic excitatory/inhibitory inputs. (**B**) Functional strength of synaptic drive in the frame of binomial model of synaptic transmission is determined presynaptically by RRP, the readily releasable pool of vesicles, and release probability, and postsynaptically by the quantal size, the amplitude of postsynaptic response to release of a single vesicle, and its kinetics (charge). Miniature postsynaptic currents (mPSCs), i.e., synaptic responses measured in the presence of tetrodotoxin, a blocker of voltage-gated Na+ channels, are often used as a quantal size estimate. Note that the RRP size is not a constant and depends of the rate of vesicle recycling, RRP refilling rate.

Various genetic mouse models of neurological diseases allow for the investigation of pathophysiological symptom manifestations over a relatively long time. In addition, it is possible to study quantitative changes in neuronal functioning using in vitro preparations, including acute brain slices. A transient E/I imbalance, supposed to be a cause of several mental disorders, may, however, reflect a physiological tool with a goal of stabilizing sensory information processing, i.e., aiming to minimize possible pathological changes induced by gene deficiency during early development [13]. Indeed, only a transient E/I imbalance has been reported in several studies; for example, [12]. If this is the case, another compensatory process might be expected to be recruited for the stabilization of information flow later during development. These mechanisms would represent the possible targets for medications that might be more selective for a specific disorder compared with the general E/I imbalance. This review summarizes only some experimental studies in which several quantitative parameters of synaptic transmission at a single cell level were obtained in the same model of a neurological disease. In most of the selected studies, measurements were performed at several developmental points, allowing us to follow not only the time profile of the E/I imbalance but also the involvement of compensatory mechanisms. For clarity, only phasic synaptic transmission will be discussed in this review, using the following parameters: the quantal size of the postsynaptic response, including its kinetics; readily releasable pool of presynaptic vesicles (RRP); RRP replenishment; the amplitude and kinetics of evoked postsynaptic responses; and presynaptic release probability (Figure 1).

## 3. Fragile X Syndrome (FXS)

Fragile X syndrome is one of the most frequent forms of inherited intellectual disability, and is often associated with autistic spectrum disorder (ASD) and attentional and social deficits (for review, [30,31]). The cause of FXS is a CGG triplet repeat expansion in the fragile X mental retardation 1 (FMR1) gene in the X chromosome [32]. The inactivation of the gene by more than 200 CGG repeats results in the absence of the encoded FMR1 protein (FMRP), an RNA-binding protein that plays an important role in the regulation of many processes in the CNS (for reviews [33,34]). At a single cell level, FMRP binds and translationally regulates mRNAs both pre- and postsynaptically, including GABAergic—both GABA_B_R [35] and GABA_A_R [36]—and glutamatergic—ionotropic [37] and metabotropic receptor [38]—subunit mRNAs. Fragile X KO mice, generated by the interruption of the *Fmf1* gene [39], demonstrate cognitive and behavioral deficits similar to those in human fragile X patients.

Developmental changes in synaptic transmission in FMR1-KO mice during postnatal development have been investigated by several groups, both in the hippocampus and cerebral cortex. Evoked (e)EPSCs induced by high-frequency electrical stimulation (2–200 Hz) show significantly potentiated responses at frequencies >20 Hz in the hippocampus of FMR1-KO mice at P15–25 [40]. No differences in the eEPSC kinetics and paired-pulse ratio (PPR) have been observed between WT and FMP1-KO mice, indicating no significant difference in either the postsynaptic receptor composition or presynaptic release probability. However, the augmentation of synaptic strength, i.e., the potentiation of the evoked response during a train of stimuli, is significantly increased in FMP1-KO mice. Both electron microscopic data and the functional RRP estimation using a high-frequency stimulation show a larger RRP size and increased reserve pool of vesicles in FMR1-KO hippocampus (also [41]), whereas the quantal amplitudes do not differ in the two phenotypes [40]. Using the FM1-43 staining/destaining protocols [42], an increased rate of vesicle recycling has been observed, i.e., a faster RRP replenishment rate. The authors suggested that the broadening of action potentials (APs) resulting from the reduced density of K^+^ channels leads to a larger Ca^2+^ influx and, therefore, faster vesicle recycling [43,44]. In summary, the increased RRP size and increased vesicle recycling rate strengthen glutamatergic transmission, shifting the E/I balance towards excitation. Data from the mPFC confirm changes in the RRP size and its replenishment in FMR1-KO mice. Interestingly, despite the reported anatomical hyper-connectivity in the mPFC of FMR1-KO mice, the mean amplitude of eEPSCs and presynaptic release probability are not significantly different in WT and FMP1-KO animals. However, the RRP refilling after short high-frequency trains is significantly slower in mutant mice compared to WT ones, showing that transient hyper-connectivity in the mPFC is compensated for by a smaller number of vesicles in the RRP and, presumably, a slower recycling of presynaptic vesicles. This functional compensation is transient and disappears by P20–36 [45].

**Figure 2:**
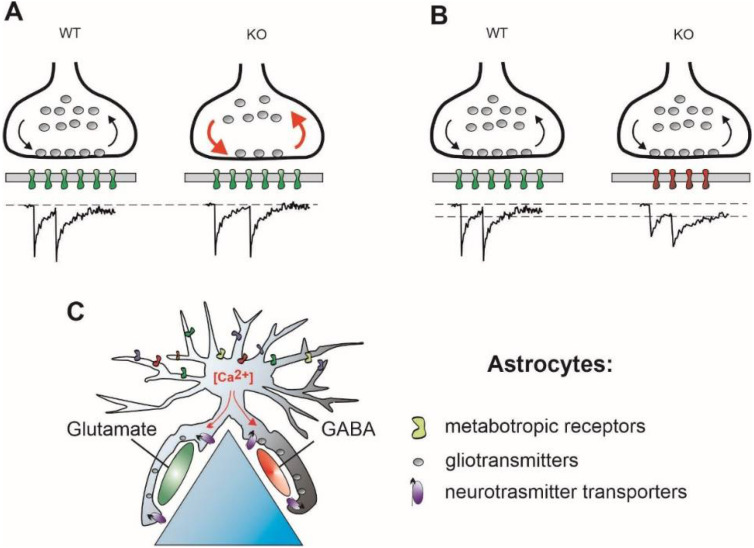
Possible mechanisms operating to correct the E/I imbalance but leading to a distorted timing of synaptic transmission. (**A**) Presynaptically, reduced RRP size may be counterbalanced by faster vesicle recycling rate, red arrows, supporting proper synaptic function during periods of activity (Mecp2-KO and FMR1-KO). Note a temporal jitter of postsynaptic responses at KO synapses. (**B**) Postsynaptically, reduced number of postsynaptic receptors, a smaller quantal size, is compensated for by involvement of slower postsynaptic receptors, stabilizing, in turn, the quantal charge (FMR1-, Nlg4-, and DTNBP1-KO). (**C**) Astrocytic ionic signaling induced by neuronal activity leads to spatially heterogeneous release of gliotransmitters and/or changes in neurotransmitter uptake rate (Nlg4-KO).

GABAergic transmission is also affected in FMR1-KO mice. In the adult (8–12 weeks) cortex, a strong (up to 50%) reduction in the expression of several subunits of GABA_A_Rs, including α1, the main adult subunit, and α3, but not α2, which is one of the main juvenile subunits ([46], for review [47]), has been reported, indicating a postsynaptic weakening of GABAergic transmission in FMR1-KO mice. In a recent study, GABAergic transmission has been investigated in the mPFC of WT and FMP1-KO mice at prepubescent (3 weeks) and adolescent (6 weeks) ages. At the 3-week age, GABAergic synaptic transmission is significantly potentiated. The quantal amplitude (the mean mIPSC amplitude) is significantly increased as a result of a higher number of postsynaptic GABA_A_Rs. Although the presynaptic release probability shows no difference between phenotypes, a larger RRP size in FMR1-KO mice attenuates the strength of tetanic depression during high-frequency stimulation [48]. Together with the above-described changes in glutamatergic transmission, these results favor the suggestion that the observed changes in GABAergic transmission might stabilize the E/I balance in FMR1-KO mice at this age. Interestingly, the above mentioned changes in GABAergic transmission are transient, i.e., the quantal amplitude and RRP size become comparable in FMR1-KO and WT mice at the 6-week age, mirroring the weakening of glutamatergic transmission [45]. However, instead of presynaptic changes, the expression of α2 subunits of GABA_A_Rs is strongly increased in FMRP-KO animals at the 6-week age. As the α2 subunit-containing GABA_A_R-mediated currents are much slower compared with the α1 subunit-containing GABA_A_R-mediated currents [17], this change strengthens GABAergic transmission postsynaptically (Figure 2). Thus, during the presymptomatic period, the E/I balance is tuned mostly by presynaptic factors, whereas, in the symptomatic age, the postsynaptic site plays the most important role. It is important to mention that, although the involvement of slower postsynaptic receptors increases the strength of the synaptic input, it decreases the temporal precision of information flow. Consequently, a specific modulation of α2,3 subunit-containing GABA_A_R-mediated transmission may be considered as a possible medical intervention [49].

## 4. Neuroligin-4 (Nlgn 4-KO)

Neuroligin-4 belongs to the neuroligin family (Nlgn1-4) of proteins, which are highly expressed throughout the CNS. Neuroligins are postsynaptic adhesion proteins that play an important role in synapse formation and function [50]. Some neuroligin isoforms are located to specific groups of synapses; for instance, Nlgn-2 is expressed exclusively at inhibitory synapses [51], indicating that loss-of-function mutations of neuroligins can directly influence the E/I balance.

In the mouse hippocampus, no specific synaptic location of Nlgn4 has been detected [52]. In addition, no difference in the number of postsynaptic density (PSD)-95 puncta, a marker of glutamatergic synapses, was revealed at perisomatic regions of pyramidal neurons, pointing to a similar number of excitatory synapses in two genotypes. The frequencies, mean amplitudes, and kinetics of mEPSC are not significantly different in WT and Nlgn4-KO neurons, indicating a similar strength of the excitatory drive in both WT and Nlgn4-KO hippocampi. However, the number of perisomatic gephyrin- and GABA_A_*γ*2-positive puncta, two postsynaptic markers of inhibitory synapses, is significantly decreased in both the juvenile (P12–26) and the adult (8–12 weeks) Nlgn4-KO hippocampus. Presynaptically, the number of vesicular inhibitory amino acid transporter (VIAAT)-positive puncta, a presynaptic marker for inhibitory synapses, remained unchanged, showing no Nlgn4-induced change at the presynaptic site. In addition, neither the mean amplitude nor frequency of mIPSCs is affected in the Nlgn-4 KO mice, confirming the absence of presynaptic Nlgn4-induced changes in GABAergic transmission. On the other hand, IPSC kinetics are significantly slowed down in terms of both the rise time and decay time constants of mIPSCs [52]. These results suggest that a reduction in the number of postsynaptic GABA_A_Rs is at least partially compensated for by slower IPSC kinetics, which, in turn, potentiates the strength of inhibition by increasing the IPSC charge (Figure 2B). However, an unchanged number of VIAAT-positive puncta does not allow for answering the question of if functional properties of presynapses are modified by a Nlgn-4 deficiency.

This question has been tackled in the work of Delattre et al. [53] performed in the mouse somatosensory cortex. In this study, evoked PSCs, both excitatory and inhibitory, were elicited by trains of electric pulses. In line with the above study [52], the kinetics of eEPSCs do not significantly differ in WT and Nlgn4-KO mice, but the mean eEPSC amplitude is reduced in Nlgn-4-KO mice. This might mean a reduction in RRP size and/or presynaptic release probability. Indeed, a reduction in the glutamatergic RRP and no change in the quantal size have been reported in the somatosensory cortex of Nlgn4-KO mice [54]. PPR, a parameter often used to estimate the release probability, is significantly increased in Nlgn4-KO mice, indicating a reduced presynaptic release probability in Nlgn-4-KO animals [53]. In summary, in Nlgn4-KO animals, the glutamatergic drive is weakened as a result of a decreased presynaptic release probability and reduced RRP size. GABAergic transmission in Nlgn-4-KO mice also demonstrates a reduced strength. The mIPSC frequency and the mean amplitude of eIPSCs are decreased in Nlgn-4-KO mice, in line with the observed reduction in RRP size. Similar to the data from glutamatergic transmission, PPR at short interstimulus intervals (25–50 ms) is significantly increased in Nlgn-4-KO mice, indicating a decreased presynaptic release probability. In line with [52], both the rise time and decay time constant of eIPSCs are significantly larger, leading to an increased charge transfer and stronger inhibition [53,54]. Interestingly, if the E/I balance was estimated as the ratio of (1) mEPSC frequency to mIPSC frequency or (2) the mean eEPSC amplitude to mean eIPSC amplitude, a significant shift in the E/I balance towards inhibition would be observed in Nlgn4-KO mice. However, because of several reported pre- and postsynaptic changes in both excitatory and inhibitory drives, the E/I ratio remains unchanged up to the high simulation frequencies (>50 Hz, [55]). As single whisker stimulation-induced responses in the somatosensory cortex of WT and Nlgn-4-KO mice do not show a significant difference [54], we might hypothesize that all observed changes are compensatory and aim to stabilize sensory information processing in the barrel cortex. On the other hand, all of the above changes fail to stabilize the intracortical/intrahippocampal information processing, revealed by the observed perturbed oscillations in the *γ*-range in the hippocampus [52] and *α*-range in the mouse barrel cortex [54]. Unfortunately, the subunit composition of GABA_A_Rs has not been directly investigated in the above cited studies. However, the observed slowing of GABA_A_R-mediated IPSCs favors the hypothesis that, also in this case, the number of slow, presumably *α*2/*α*3, subunit-containing GABA_A_Rs is increased in Nlgn-4 KO cells, pointing to a disturbed timing of information flow and thus providing a possible target for therapeutic medication (Table 1).

## 5. Rett Syndrome (RTT)

Rett syndrome (RTT) is a genetic neurodevelopmental disorder associated with intellectual deficits and autistic behavior that predominantly occurs in females. In most cases, RTT is linked to an abnormal function of a single gene, the methyl-CpG-binding protein 2 (MECP2), on the X chromosome. Affected individuals begin to develop normally but regress after 6 to 18 months. Mecp2-deficient mice demonstrate normal behavior till the fifth postnatal week and start to exhibit behavioral abnormalities similar to the ones observed in individuals with RTT thereafter [55,65].

Electrophysiological recordings in cortical brain slices reveal a reduced spontaneous firing of L5 pyramidal neurons in Mecp2-KO mice, although their intrinsic passive and active membrane properties are not significantly changed. However, despite the fact that the onset of typical RTT symptoms is after 4–5 postnatal weeks, the spontaneous firing of L5 neurons is already reduced at 2–3 weeks of age, i.e., preceding the symptomatic age. Interestingly, mean frequencies of both mEPSCs and mIPSCs do not differ in WT and Mecp2-KO mice, indirectly indicating no difference in the RRP size and release probability. Thus, the E/I ratio, defined as a ratio of the mEPSC frequency to mIPSC frequency, demonstrates no imbalance. Postsynaptically, however, the mean mEPSC amplitude is significantly reduced, indicating a decrease in excitatory strength as a result of a reduced number of postsynaptic receptors. The mean mIPSC amplitudes do not demonstrate any significant difference between genotypes. Thus, the E/I ratio, when defined as the ratio of excitatory to inhibitory charge transfers, reveals a strong shift towards inhibition. Interestingly, the authors also reported that both the rise and decay time constants of mIPSCs are significantly shortened in Mecp2-KO mice, pointing to a decrease in an individual mIPSC charge. Thus, in contrast to the aforementioned Nlgn4-KO mice, where IPSCs have been slowed down to potentiate inhibition, in Mecp2-KO mice, IPSCs are faster in order to decrease the excess inhibition, increasing, in parallel, the temporal precision of inhibitory transmission at this presymptomatic age. Unfortunately, the IPSC kinetics and/or subunit composition of GABA_A_Rs are not discussed in the cited work [56].

Lo et al. [57] specifically investigated the E/I balance in layer four spiny stellate neurons in thalamocortical slices of the barrel cortex in 3–5 weeks old mice, i.e., at the symptomatic age. Experiments showed a potentiated GABAergic feedforward inhibition and a reduced E/I ratio of thalamocortical responses. Using the paired-pulse protocol, a similar mean amplitude and PPR of eEPSPs have been reported, indicating that the presynaptic release probability is not affected by Mecp2 deficiency. Although the authors did not directly estimate the RRP, the number of glutamatergic and GABAergic projections has been reported to be comparable in WT and Mecp2-KO mice. The mEPSC and mIPSC frequencies are not significantly different between genotypes, supporting the idea that the presynaptic properties of both glutamatergic and GABAergic synaptic contacts are not modified by Mepc2 deficiency. Postsynaptically, however, the mIPSC amplitude is larger in Mecp2-KO animals compared with WT ones, indicating a strong increase in the quantal charge. While the authors did not report IPSP kinetics to compare the possible reduction in inhibition, they suggest the involvement of slow α2/α3-containg GABA_A_Rs [57]. Thus, both at presymptomatic and symptomatic ages, several tuning(s) of synaptic strength take place that stabilize the E/I ratio; in this case, mainly through postsynaptic changes. 

In the hippocampus of Mecp2 mutants, properties of synaptic transmission have been investigated in presymptomatic (about P25) and symptomatic (>P40) mice using optical and electrophysiological approaches. Using a voltage sensitive dye, an overall decrease in network excitability has been detected, but this alteration is, however, independent of the genotype of animals. Extracellular MUA recordings at the symptomatic stage revealed a higher spontaneous activity in Mecp2-KO animals. The number of both glutamatergic and GABAergic synapses is reported to be unchanged, but the number of docked vesicles, i.e., the RRP size, is reduced at both excitatory and inhibitory synapses in a similar way to approximately 60% of the number of WT synapses. Applying a paired-pulse protocol, a decreased PPR is detected in Mecp2 mutants, indicating a higher presynaptic release probability. Despite the smaller number of vesicles in the RRP and higher release probability, the rate of RRP depletion, as measured by means of FM1-43, is significantly slowed down, indicating a faster refilling of the RRP [58]. Unfortunately, extracellular recordings do not allow us to study postsynaptic responses. Summarizing the data from this work, the reduced RRP size is counterbalanced by the faster RRP refilling rate, thus stabilizing the E/I ratio during a period of activity (Figure 2A). Postsynaptic modifications induced by Mecp-2 deficiency have been studied in the subsequent work. Using an electrophysiological approach, mIPSCs and mEPSCs have been recorded in the hippocampus of WT and Mecp-2-KO mice. The mean mIPSC frequency is higher, whereas the frequency of mEPSCs shows a reduction in Mecp-2-KO mice. Thus, the E/I ratio, defined as a ratio of the mEPSC frequency to mIPSC frequency, demonstrates a significant shift towards inhibition. However, the mean mIPSC amplitude and, in turn, the mean mIPSC charge is reported to be decreased in Mecp2-KO mice, whereas both the mean mEPSC amplitude and mEPSC charge show an increase. As a consequence, if the E/I ratio is defined as the ratio of mEPSC charge to mIPSC charge transfer, a significant E/I ratio shift towards excitation becomes obvious. Immunohistochemically, staining with antibodies against the α1 subunit of GABA_A_Rs reveals significantly smaller α1-positive puncta, which is in line with the smaller mIPSC amplitude. In addition, the mean amplitude of eIPSCs is significantly smaller in the Mecp-2-KO mice, suggesting a smaller RRP size and/or release probability. Unfortunately, neither the RRP refilling rate nor PSC kinetics have been analyzed in this study [59]. It can be only hypothesized that the presynaptic reduction in RRP size is counterbalanced by a faster RRP refilling rate, and the smaller number of postsynaptic receptors is compensated for by their slower kinetics (Table 1). These changes might contribute to the E/I stabilization, but they have the potential to distort the fast timing of information transfer (Figure 2A,B).

## 6. Oligophrenia

Oligophrenia is another X-linked mental retardation disorder. A loss-of-function mutation of the *Ophn1* gene, which encodes oligophrenin-1 protein, leads to intellectual and learning disabilities both in humans and mice [66,67]. On the cellular level, oligophrenin-1 dysfunction affects several morphological and functional parameters, including spine morphology, maturation of synapses, rate of vesicle recycling, stability of postsynaptic AMPA receptors, and long-term plasticity of excitatory transmission [68].

Power et al. [60,61] performed an extensive quantitative study of both excitatory and inhibitory synaptic transmissions in the hippocampus of Ophn-1-deficient mice. No differences in passive and active intrinsic properties have been found between phenotypes in the dentate gyrus granule neurons or CA3 pyramidal neurons. In line with the reduced spine density observed anatomically, the mean amplitude of eEPSCs is significantly smaller in Ophn1-KO mice. Whereas the quantal amplitude remains constant, the frequency of sEPSCs is strongly decreased in Ophn1-KO mice, indicating a change in the RRP size and/or release probability. Thus, Ophn1 deficiency leads to the presynaptic weakening of the glutamatergic drive.

Inhibitory transmission demonstrates similar changes—a reduced amplitude of eIPSCs, lower frequency of sIPSCs, and no difference in the sIPSC amplitude—confirming the presynaptic locus of the effects induced by Ophn1 dysfunction at GABAergic synapses. Although the frequency of mIPSCs is not different between genotypes, both the application of hyperosmotic solution and high-frequency stimulation reveals a significantly reduced RRP size in Ophn1-KO mice. Nevertheless, the mean amplitude of minimal stimulation-induced eIPSCs, i.e., the stimulation of a single axon, is not dependent on the phenotype, indicating a higher release probability in Ophn1-KO animals. These two presynaptic changes—a higher release probability and smaller RRP size—may potentially lead to a faster depletion of the presynaptic poll of vesicles, i.e., a high-frequency synaptic fatigue. However, Ophn1-KO GABAergic synapses do not demonstrate a significant depression of synaptic transmission in response to high-frequency stimulation; the mean amplitude of eIPSCs remains stable during a train of stimuli [61]. At the same time, excitatory connections show a strong depression during high-frequency stimulation [61]. These data suggest that the recycling rate of presynaptic vesicles is modulated by presynaptic activity and that this tuning differs at glutamatergic and GABAergic terminals, resulting in a stable E/I ratio at physiologically relevant levels of network activity. Therefore, presynaptic Ca^2+^ signaling, as a factor that influences the recycling rate of vesicles [26], appears to be a promising target to be investigated in the case of Ophn-1 deficiency. 

## 7. Schizophrenia

Schizophrenia is a neuropsychiatric disease characterized by specific symptoms, cognitive deficits, and social behavior impairments. An increasing amount of evidence gives rise to the hypothesis that synaptic transmission dysfunctions, including dopaminergic, glutamatergic, and GABAergic ones, are the main cause of schizophrenia symptomatology (for recent reviews, [69,70]. Despite the discovery of several genes whose mutations are associated with this disease (for review, [71,72]), the precise etiology of schizophrenia remains poorly understood. Here, only one mouse model, in which, quantitative investigations of glutamatergic and GABAergic transmissions have been performed, will be discussed.

One of the schizophrenia-susceptibility genes is the dystrobrevin-binding protein 1 (DTNBP1). This gene encodes the dysbindin protein that interacts with some SNARE proteins and can thus directly influence synaptic transmission. In the CA1 area of the hippocampus of DTNBP1-KO mice, the quantal size, determined as the mean mEPSC amplitude, is not changed, whereas the mEPSC frequency shows a significant decrease. In line with the latter result, the RRP size, estimated by means of electron microscopy and functionally by electrical stimulation, is decreased in DTNBP1-KO animals. The rate of RRP replenishment is not significantly different between genotypes. As a consequence, the mean eEPSC amplitude is significantly decreased in DTNBP1-KO mice. Interestingly, the rise and decay time constants of both mEPSCs and eEPSCs show a significant increase. Consequently, despite the reduced mean eEPSC amplitude in DTNBP1-KO mice, the eEPSC charge transfer is comparable between WT and DTNBP1-KO mice [62]. Thus, the presynaptic weakening of the excitatory drive in DTNBP1-KO animals is counterbalanced by the postsynaptic site, keeping at least the strength of excitatory inputs constant.

In the prefrontal cortex of DTNBP1-deficient animals, both the mean mEPSC amplitude and mEPSC frequency are decreased compared with WT ones, showing that the genetic inactivation of DTNB1 induces both pre- and postsynaptic changes. The decrease in PPR observed in DTNBP1-KO mice gives support to the hypothesis that the presynaptic release probability is increased in mutants. Consequently, the mean eEPSC amplitude is decreased in DTNBP1-KO mice compared with WT mice [63]. Unfortunately, EPSC kinetics were not analyzed in this work. In summary, although the presynaptic release probability is increased, the smaller quantal size results in a weakening of the excitatory drive in DTNBP1-KO mice.

The effects of dysbindin dysfunction on the GABAergic transmission in the prefrontal cortex have been investigated in another study [64]. Recordings from pyramidal neurons showed that the mean amplitude of mIPSCs is significantly decreased, but the mIPSC kinetics become slower in DTNBP1-KO mice, making the quantal charge transfer comparable in both phenotypes. Although PPR has not been investigated in the study, an increase in the sIPSC frequency in DTNBP1-KO mice suggests an increase in the presynaptic release probability. Taken together, these data show that, in a DTNBP1-KO mouse model, the postsynaptic weakening of both glutamatergic and GABAergic transmission, presumably due to a reduction in the number of receptors, is partially compensated for by the slower kinetics of postsynaptic responses and a higher presynaptic release probability (Figure 2B, Table 1). Thus, in this mouse model, both the presynaptic vesicle release and postsynaptic receptors might be suggested as a target for medical intervention.

## 8. Glia-Mediated Control of E/I Balance

There is increasing evidence that the functional synaptic strength is determined not only by pre- and postsynaptic neurons but also by glial cells. Astrocytes may modulate both short-term and long-term plasticity by various mechanisms, including the rate of neurotransmitter clearance or release of gliotransmitters that can directly influence synaptic efficacy. For example, in the plasticity-related gene 1(PRG1)-KO mice, a schizophrenia mouse model, hyperexcitability in the hippocampus and cerebral cortex results from a selective potentiation of glutamatergic transmission [73,74]. PRG1 is located presynaptically to glutamatergic terminals and neutralizes lysophosphatidic acid (LPA), which modulates the presynaptic release probability of glutamate through LPA_2_ receptors. LPA is synthesized by autotaxin (ATX), which is expressed selectively in astrocytic processes. Furthermore, its enzymatic activity is regulated by neuronal activity detected and converted into intra-astrocytic ionic signaling via astrocytic glutamate receptors. This indicates that astrocytes may, locally, at the single synapse level, control the strength of the glutamatergic drive, and, in turn, the E/I balance ([75]; for review of the role of astrocytes in schizophrenia, [76]). Similarly, the release of D-serine from astrocytes selectively facilitates glutamatergic transmission, shifting the E/I balance towards excitation [77]. Furthermore, the potentiation of astrogenesis during embryonic development selectively affects the strength of glutamatergic transmission and results in ASD symptoms in adult animals. The increase in the mean amplitude of eEPSCs without a matched change in the mIPSC amplitude points to an increased number of synaptic contacts and/or the RRP of presynaptic vesicles as the most probable mechanism mediating the observed changes [78].

In Tsc2+/− mice, an ASD mouse model, the E/I balance in the mPFC, determined as the ratio of mEPSC frequency to mIPSC frequency, is controlled by the activity of presynaptic GABA_B_Rs [79]. Unexpectedly, the tonic GABA_B_R-mediated inhibition of GABAergic synapses, but not glutamatergic ones, is significantly stronger in Tsc2+/− animals compared with WT ones. As no difference in the functional properties of GABA_B_Rs in WT and Tsc2+/− mice has been detected, this difference in GABA_B_R-mediated tonic inhibition is most probably established by the level of the local ambient GABA concentration. As the extracellular GABA level is controlled by GABA transporters, which are predominantly located to astrocytes, and, additionally, astrocytes are capable of GABA release ([80,81,82], astrocytic ionic homeostasis might shift the E/I balance locally depending on the current level neuronal activity. Astrocytes can measure the latter by means of a variety of metabotropic receptors, converting neuronal activity into intracellular Ca^2+^ and Na^+^ signaling (Figure 2C, [83,84]).

In addition to GABA and D-serine, astrocytes are capable of ATP release [85]. Intra-astrocytic Ca^2+^ transients elicited by Ca^2+^ release from the intracellular stores stimulate an astrocyte-mediated ATP release that contributes to synapse elimination [86]. In addition, adenosine, a product of the metabolic degradation of ATP, alleviates the hyperexcitability of hippocampal networks in the mouse model of Rett syndrome [58]. Surprisingly, the astrocyte-mediated release of ATP followed by adenosine A2 receptor activation influences both the generation and propagation of action potentials in neurons, affecting, in this way, at least the timing of information flow in the CNS [87]. Despite intensive experimental efforts, spatial aspects of both astrocytic ionic signaling and gliotransmitter release are still elusive (for a recent review, [88]). Therefore, astrocytes appear to represent a promising, although not yet thoroughly investigated, target for the tuning of the distorted timing of synaptic transmission and information flow and, in turn, a target for the medical treatment of neurological disorders (Figure 2C).

## 9. Conclusions

In this review, quantitative data on both excitatory and inhibitory synaptic transmission obtained in several genetic mouse models of neurological diseases have been analyzed and compared. Although a change in the E/I ratio appears to be a hallmark of various diseases, the precise mechanisms that lead to an E/I imbalance are missing. Even the physiological meaning of an E/I shift remains elusive because the comparisons performed using different methodological approaches—for example, the number of spines, cells, receptors, etc.—are sometimes contradictory. In this review, the number of used parameters has been reduced by applying the binomial model of synaptic transmission, thus allowing for a direct comparison across a broad range of mouse models. Interestingly, a transient E/I imbalance seems to be frequently counterbalanced by another process, and such compensation mechanisms have been observed in several unrelated mouse models. A decrease in the presynaptic pool of synaptic vesicles is usually accompanied by a faster, presumably Ca^2+^-dependent recycling of presynaptic vesicles. Thus, a smaller response to a single action potential is counterbalanced by a faster refilling rate, enabling, in turn, a stable synaptic transmission during high levels of activity. Such compensation can rescue the mean strength of the presynaptic release, but will probably distort the timing of the action-potential-mediated release. Treatments affecting presynaptic voltage-gated Ca^2+^ channels might be considered as a potential medication target. Postsynaptically, a decreased quantal size, usually as a result of a smaller number of postsynaptic receptors, is frequently compensated for by the replacement of fast postsynaptic receptors with slower ones, thus supporting a temporal summation and thus stabilizing the charge transfer, i.e., the functional strength of the synaptic drive. Thus, treatments that can influence single channel properties and/or kinetics of postsynaptic receptors may be taken into consideration. The presented analysis gives support to the hypothesis that a transient E/I imbalance during early development might be a stabilizing factor that aims to lessen the functional effects of gene deficiency-induced changes and to enable a physiologically “correct” information flow during a limited time window. Later in development, the disturbed E/I ratio is balanced by means of both pre- and postsynaptic changes, but this rescue of the “mean” E/I balance leads to a partially distorted timing of information transfer.

## Figures and Tables

**Table 1:** Changes in glutamatergic and GABAergic transmission observed in the mouse models. Empty boxes mean “no data reported”. Note that both mean amplitude and area of mPSCs (if reported) are mentioned in the column “Quantal size”.

Mouse Model (Disease)	Excitation/ Inhibition	RRP	Pv	Quantal Size	RRP Refilling	References
FMP1-KO (Fragile X)	Glutamate	Increased	Not changed	Not changed	Accelerated	[40,42]
	GABA	Increased	Not changed	Charge increased		[47,48]
Nlgn-4-KO (Autistic spectrum disorder)	Glutamate	Reduced	Reduced	Not changed		[52,53]
	GABA	Reduced	Reduced	Charge increased		[53,54]
Mecp2-KO (Rett syndrome)	Glutamate	Not changed Reduced	Not changed Increased	Reduced		[56,57] [58]
	GABA	Not changed Reduced	Not changed	Amplitude—increased, Charge —reduced	Accelerated	[56,57] [59]
Ophn1-KO (Oligophrenia)	Glutamate	Increased		Not changed	Slowed	[60,61]
	GABA	Reduced	Increased	Not changed	Accelerated	[61]
DTNBP1-KO (Schizophrenia)	Glutamate	Reduced	Increased	Amplitude—not changed, Charge—increased	Not changed	[62,63]
	GABA		Increased	Amplitude—decreased, Charge—not changed		[64]

## Data Availability

Not applicable.
